# Semi-Synthetic Click-Gelatin Hydrogels as Tunable Platforms for 3D Cancer Cell Culture

**DOI:** 10.3390/gels8120821

**Published:** 2022-12-12

**Authors:** Luke Hipwood, Julien Clegg, Angus Weekes, Jordan W. Davern, Tim R. Dargaville, Christoph Meinert, Nathalie Bock

**Affiliations:** 1 Faculty of Health, School of Biomedical Sciences, Queensland University of Technology (QUT), Brisbane, QLD 4059, Australia; 2 Centre for Biomedical Technologies, Science and Engineering Faculty, School of Mechanical, Medical and Process Engineering, Queensland University of Technology (QUT), Brisbane, QLD 4059, Australia; 3 Gelomics Pty Ltd., Brisbane, QLD 4059, Australia; 4 Centre for Materials Science, Faculty of Science, School of Chemistry and Physics, Queensland University of Technology (QUT), Brisbane, QLD 4000, Australia; 5 Australian Prostate Cancer Research Centre (APCRC-Q), Queensland University of Technology (QUT), Brisbane, QLD 4059, Australia; 6 Max Planck Queensland Centre (MPQC), Queensland University of Technology (QUT), Brisbane, QLD 4059, Australia; 7 Translational Research Institute (TRI), Brisbane, QLD 4102, Australia

**Keywords:** gelatin, hydrogels, 3D cancer model, extracellular matrix, click chemistry, cell culture

## Abstract

Basement membrane extracts (BME) derived from Engelbreth–Holm–Swarm (EHS) mouse sarcomas such as Matrigel^®^ remain the gold standard extracellular matrix (ECM) for three-dimensional (3D) cell culture in cancer research. Yet, BMEs suffer from substantial batch-to-batch variation, ill-defined composition, and lack the ability for physichochemical manipulation. Here, we developed a novel 3D cell culture system based on thiolated gelatin (Gel-SH), an inexpensive and highly controlled raw material capable of forming hydrogels with a high level of biophysical control and cell-instructive bioactivity. We demonstrate the successful thiolation of gelatin raw materials to enable rapid covalent crosslinking upon mixing with a synthetic poly(ethylene glycol) (PEG)-based crosslinker. The mechanical properties of the resulting gelatin-based hydrogels were readily tuned by varying precursor material concentrations, with Young’s moduli ranging from ~2.5 to 5.8 kPa. All hydrogels of varying stiffnesses supported the viability and proliferation of MDA-MB-231 and MCF-7 breast cancer cell lines for 14 and 21 days of cell culture, respectively. Additionally, the gelatin-based hydrogels supported the growth, viability, and osteogenic differentiation of patient-derived preosteoblasts over 28 days of culture. Collectively, our data demonstrate that gelatin-based biomaterials provide an inexpensive and tunable 3D cell culture platform that may overcome the limitations of traditional BMEs.

## 1. Introduction

Three-dimensional (3D) models are advantageous systems for cell culture due to their ability to replicate biochemical, physical, spatial, and temporal properties of the cellular microenvironment as seen in vivo [1,2,3]. For 3D models, in vivo properties of the cellular microenvironment can be matched using physiologically relevant biomaterial constituents, which can form bioactive structures. These structures possess biophysical properties, such as mechanical strength and cell adhesion sites, similar to the in vivo extracellular matrix (ECM) [4,5,6,7]. The composition of the in vivo ECM is tissue-specific, as the amount and structural organization of ECM molecules vary between tissues; however, these molecules are primarily proteins, glycoproteins, proteoglycans and polysaccharides [8].

In contrast, traditional two-dimensional (2D) models utilize flat, highly stiff, physiologically inert materials such as glass or plastic for cell propagation [9,10,11]. 2D models are unable to facilitate cell signaling pathways as they would occur in vivo, as signaling in 2D models is restricted to the *x–y* plane, whereas cells in vivo could receive signals in all three dimensions [11,12]. Additionally, 2D models do not possess the ECM molecule-rich environment of the in vivo ECM. These conditions cause cells grown in 2D to exhibit altered and unnatural phenotypic expression [11], leading to a lack of translation between 2D cell culture models and clinical trials [13,14]. Therefore, many methods for 3D cell culture have been developed, with the intention of providing cell culture platforms that more accurately recapitulate the in vivo ECM, and can translate preclinical results to desirable clinical outcomes [15,16]. Currently, the gold standard for 3D cell culture models is basement membrane extracts (BMEs) derived from Engelbreth–Holm–Swarm mouse sarcomas, such as Matrigel^®^ (Corning, New York, NY, USA) [17,18,19]. BME matrices often suffer from a lack of mechanical tuneability, batch-to-batch variation, and difficult handling procedures [18,20]. For example, the standard operating procedure for encapsulation of cells in Matrigel^®^ (Corning, New York, NY, USA) matrices requires all materials to be pre-thawed at 4 °C at least 8 h prior to encapsulation, and all steps during encapsulation must be conducted at 4 °C, otherwise matrices have the potential to degrade or form a hydrogel network prior to cell encapsulation [21].

The limitations demonstrated by BME matrices have led to the development of alternative 3D cell culture platforms, which aim to address these limitations. For example, hydrogels are water-swollen polymeric networks, formed through the interaction of hydrogel precursors, that can provide suitable environments for 3D cell culture [22,23]. Poly(ethylene)-glycol (PEG)-based hydrogels are synthetic 3D cell culture matrices, produced through crosslinking of PEG-based hydrogel precursors [16,21,24,25] that can be produced with high batch-to-batch reproducibility, therefore addressing the cross-batch variability observed in BME matrices. Additionally, some PEG-based hydrogels can be mechanically tuned through variation of hydrogel precursor concentration [26]. However, PEG-based hydrogels often require functionalization with cell-instructive and bioactive motifs, such as arginylglycylaspartic acid (RGD) sequences, for cell culture to be successful [25]. The need to introduce additional motifs to synthetic matrices can prove tedious and costly, as optimization of these factors may be required to achieve the desired level of cytocompatibility.

In contrast to synthetic-based matrices, naturally derived biomaterials are inherently biocompatible and possess cell-instructive motifs that can promote cell-matrix and cell-cell communication [27], therefore eliminating the need to introduce additives. The naturally derived biomaterial gelatin has shown great promise for generation of 3D cell culture matrices, due to its inherent ECM-like properties, and versatile potential for functionalization, because of its amine and carboxylic acid-rich backbone [28]. Gelatin is produced through the denaturation and hydrolyzation of the ECM-native protein, collagen, from animal tissues, such as bovine, porcine and piscine [29,30]. Formulations consisting solely of gelatin can form gels; however, these gels are unstable matrices. This method of gel synthesis is the result of collagen-like triple-helix formation, a temperature-dependent process that results in gels that are readily degraded through temperature fluctuations [31,32]. Additionally, these gels cannot be mechanically tuned, which is important for matching hydrogel stiffness to that of in vivo tissues [4,33,34]. Therefore, gelatin must be functionalized with crosslinkable functional groups that can form stable chains between molecules within the final cell culture matrix.

Gelatin has previously been functionalized with methacryloyl [27,35] and norbornene [36] functional groups to form cytocompatible matrices via photocrosslinking, which have been heavily used in applications such as tissue engineering [37,38,39], wound healing [40,41,42], and drug delivery [43,44]. Although photocrosslinking can produce highly cytocompatible and mechanically tunable gelatin-based hydrogels [45], their method of preparation is constrained by the need for photoinitiator solutions and light sources for hydrogels to be synthesized. These constraints can make hydrogel preparation tedious.

Therefore, other crosslinking strategies, such as click-chemistry crosslinking, have been employed to produce hydrogels with improved ease-of-use [46,47,48]. Click-based crosslinking only requires two hydrogel precursors, omitting the need for light sources or photoinitiators. Thiols (-SH) can undergo Michael-type click crosslinking with maleimide (MAL) groups to form hydrogels near-instantaneously [25,49]. The SH/MAL crosslinking has previously been used with PEG-based formulations; however, the use of thiolated gelatin (Gel-SH) in the context of cell culture remains largely unexplored.

Here, we developed and characterized Gel-SH from fish gelatin, a novel, low-viscosity biomaterial, and Gel-SH/4-armed PEG-maleimide (PEG-4MAL) hydrogels, formed through Michael-type addition of Gel-SH and PEG-4MAL. We present the processing and full materials characterization involving rheological and mechanical data of Gel-SH and Gel-SH/PEG-4MAL hydrogels. Gel-SH/PEG-4MAL hydrogels possessed mechanical properties suitable for 3D breast cancer cell culture and primary bone cells, which could be tuned based on hydrogel precursor concentrations. To our knowledge, this is the first report to introduce fish-derived Gel-SH as an easy-to-use, effective, and reproducible platform for 3D cell culture of cell lines and primary cells.

## 2. Results and Discussion

**Figure 1:**
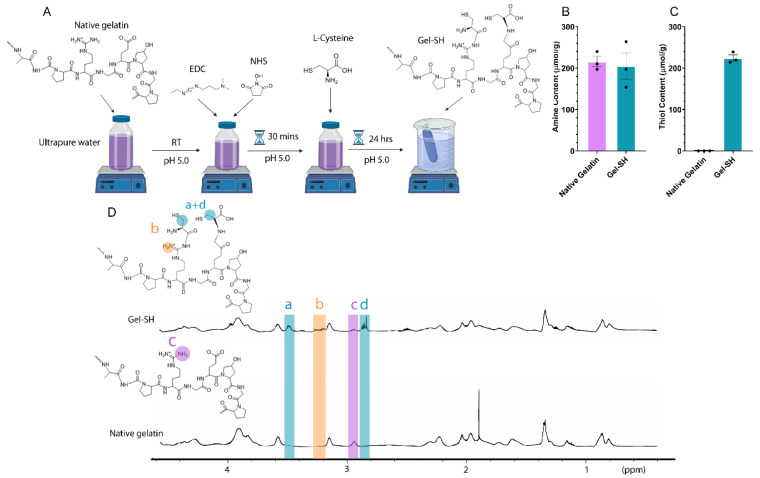
Thiolated gelatin (Gel-SH) functionalization. (**A**) Gel-SH synthesis schematic. Gel-SH was synthesized through 1-ethyl-3(3-dimethylamino)propyl carbodiimide (EDC)/*N*-hydroxysuccinimide (NHS) conjugation of L-cysteine to the native fish gelatin backbone. (**B**) Amine content of Gel-SH determined via TNBS assay (mean ± SEM; *n* = 3); (**C**) Thiol content of Gel-SH determined via DTNB assay (mean ± SEM; *n* = 3); (**D**) ^1^H-NMR of Gel-SH and native gelatin. Gelatin thiolation indicated by increase in signal at peaks (a), (b) and (d), and decrease in signal (c) in Gel-SH compared to native gelatin.

The potential of Gel-SH as a biomaterial for 3D cell culture purposes has been largely unexplored. This has most likely been because until now, the reported Gel-SH materials have been produced using mammalian-derived gelatins [50,51,52], that can be difficult to handle, and could make the preparation of Gel-SH-based hydrogels tedious. Therefore, we first aimed to develop a Gel-SH biomaterial using fish gelatin as the raw material, to overcome handling limitations associated with the highly viscous, shear-thinning nature of mammalian-derived gelatin solutions. The native gelatin backbone possesses both carboxylic acid and amine groups, therefore, we functionalized gelatin through 1-ethyl-3(3-dimethylamino)propyl carbodiimide (EDC)/*N*-hydroxysuccinimide (NHS) conjugation. EDC/NHS conjugation relies on the conjugation of either *o*-acylisourea esters, produced through EDC activation of carboxylic acids, or NHS, an amine-reactive, *o*-acylisourea ester stabilizing agent, with amine groups, and is commonly used in molecular synthesis and protein immobilization [53,54,55,56]. L-cysteine is a thiolated amino acid that also possesses carboxylic acid and amine groups. Therefore, it was hypothesized that L-cysteine could be grafted to the fish gelatin backbone through EDC/NHS conjugation, to produce a thiolated Gel-SH product (Figure 1A).

There were no significant differences in amine content observed between native fish gelatin (~216 μmol/g) and Gel-SH (~205 μmol/g) (Figure 1B), most likely due to L-cysteine possessing an amine group, resulting in a near 1:1 substitution of amines once functionalized. Native fish gelatin does not possess traceable thiol, therefore, successful thiolation was first determined through 5,5′-dithio-bis-[2-nitrobenzoic acid] (DTNB) assay (Figure 1C). The degree of thiolation influences the amount of Gel-SH and PEG-4MAL materials required to reach an equimolar (SH:MAL) ratio, which is optimal for hydrogel formation [57,58]. With increased degree of Gel-SH thiolation, there is a higher number of click bonds between Gel-SH and PEG-4MAL that can occur using the same concentration of Gel-SH, and therefore more stability in the matrix. Additionally, successful thiol functionalization was observed through comparison of the ^1^H-NMR spectra of native gelatin and Gel-SH (Figure 1D). In Gel-SH, there is a reduction in lysine-associated peaks (2.9 ppm, c in Figure 1D) as a result of molecular functionalization, which has previously been observed by Li et al. [59] and Gockler et al. [60], where gelatin was thiolated using EDC/NHS L-cysteine conjugation and *N*-homocysteine thiolation, respectively. New peaks were also observed in Figure 1D in the Gel-SH spectra at 2.8 ppm (d) and 3.5 ppm (a), associated with peaks present in other Gel-SH formulations [59,61]. An additional peak was observed at 3.2 ppm (b), which is indicative of L-cysteine amine group peaks present in Gel-SH [62]. These results showed that L-cysteine had successfully conjugated to gelatin, producing a thiolated Gel-SH molecule.

Once Gel-SH functionalization had been confirmed, rheological testing was conducted to compare the flow behavior of Gel-SH solutions to native porcine gelatin solutions. Mammalian-based gelatins, such as porcine, possess high viscosity, and shear-thinning flow behavior at room temperature (~25 °C) [63,64,65,66], therefore limiting their ease-of-use. Mammalian-based gelatin solutions are commonly warmed above their sol–gel temperature (35 °C) [29], to decrease viscosity so that they can be pipetted precisely [35]. In contrast, the Gel-SH biomaterial presented here is derived from gelatin from cold-water fish skin, which possesses a lower amount of proline and hydroxyproline amino acids [29,65,67], and therefore, a sol–gel transition temperature of ~15 °C [29], and low viscosity at room temperature. This is because proline and hydroxyproline are responsible for the formation of collagen-like triple helices, that cause gelation to occur at sol–gel transition temperatures [29,32,68]. To our knowledge, the Gel-SH described here is the first low-viscosity Gel-SH biomaterial reported, that can be readily handled at room temperature, and does not require tedious preparation methods such as heating above 35 °C prior to use. Therefore, it was hypothesized that our novel Gel-SH would possess low viscosity compared to porcine gelatin solutions, with Newtonian-like flow behavior, as such characteristics have previously been observed in solutions comprised solely of gelatin from cold-water fish skin [29,65,69].

**Figure 2:**
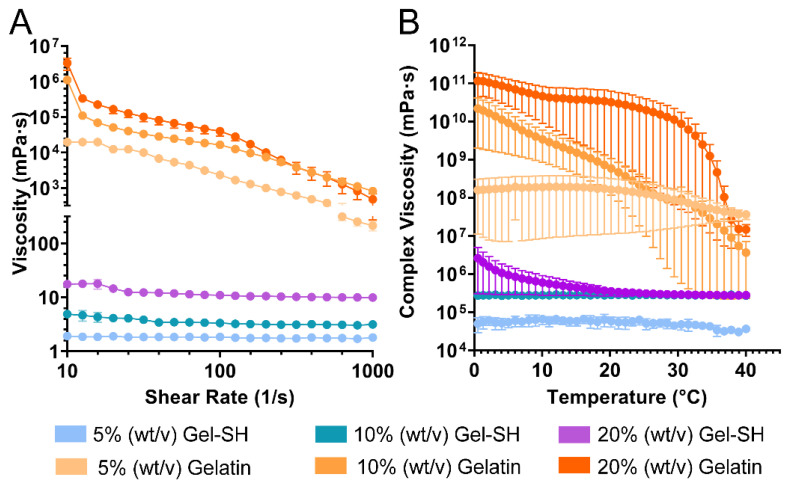
Flow properties of Gel-SH. (**A**) Shear-rate sweeps of Gel-SH and porcine gelatin were conducted at room temperature (25 °C) using a 25 mm cone plate, with shear-rate range of 0.1–1000/s, at a constant frequency of 1 Hz (mean ± SEM; *n* = 3); (**B**) Temperature sweeps of Gel-SH and porcine gelatin conducted using a 25 mm parallel plate at a constant frequency of 1 Hz, and constant strain of 1%, with temperature ramping linearly from 37 °C, at a rate of 2 °C/min (mean ± SEM; *n* = 3).

As seen in Figure 2A, at room temperature, 20% (*w*/*v*), 10% (*w*/*v*) and 5% (*w*/*v*) Gel-SH solutions all exhibited Newtonian-like flow behavior, as solution viscosities were not dependent on shear-rate. In contrast, all porcine gelatin solutions exhibited shear-thinning flow behavior, as solution viscosities decreased with increasing shear-rate. Gel-SH solutions possessed low viscosities relative to the porcine gelatin solutions, with Gel-SH solutions possessing average viscosities of ~1 mPa·s at 5% (*w*/*v*), ~3 mPa·s at 10% (*w*/*v*), and ~12 mPa·s at 20% (*w*/*v*) across the shear-rate sweep, whilst porcine gelatin solutions possessed average viscosities of ~5855 mPa·s at 5% (*w*/*v*), ~74,759 mPa·s at 10% (*w*/*v*), and ~220,000 mPa·s at 20% (*w*/*v*).

Additionally, temperature sweeps (Figure 2B) were conducted to further investigate the differences in flow behavior between Gel-SH and native porcine gelatin solutions as function of temperature. Porcine gelatin solutions possessed high complex viscosity compared to Gel-SH solutions throughout the temperature sweeps. Gel-SH solutions of 5% (*w*/*v*) and 10% (*w*/*v*) possessed no significant fluctuations in complex viscosity throughout the temperature sweeps, however, the viscosity of 20% (*w*/*v*) Gel-SH solutions began to increase within the 10–15 °C temperature range and continued to increase as temperature decreased. This increase in complex viscosity corresponds to the melting temperature of gelatin from cold-water fish skin as reported previously [65]; therefore, handling of 20% (*w*/*v*) Gel-SH solutions at temperatures below 15 °C may be more difficult than 10% (*w*/*v*) and 5% (*w*/*v*) Gel-SH solutions. In contrast, the complex viscosity of porcine gelatin solutions was dependent on temperature, as complex viscosity increased with decreasing temperature for all concentrations tested. Overall, these results demonstrate the potential for Gel-SH as a readily handleable biomaterial, as solutions do not require heating prior to use, as is the case in mammalian-based gelatin hydrogel solutions, or kept on ice throughout the procedure, as observed in Matrigel^®^ (Corning, New York, NY, USA) matrices. The Newtonian-like flow behaviour of Gel-SH solutions at room temperature is indicative of their ability to be easily handled at mild conditions.

**Figure 3:**
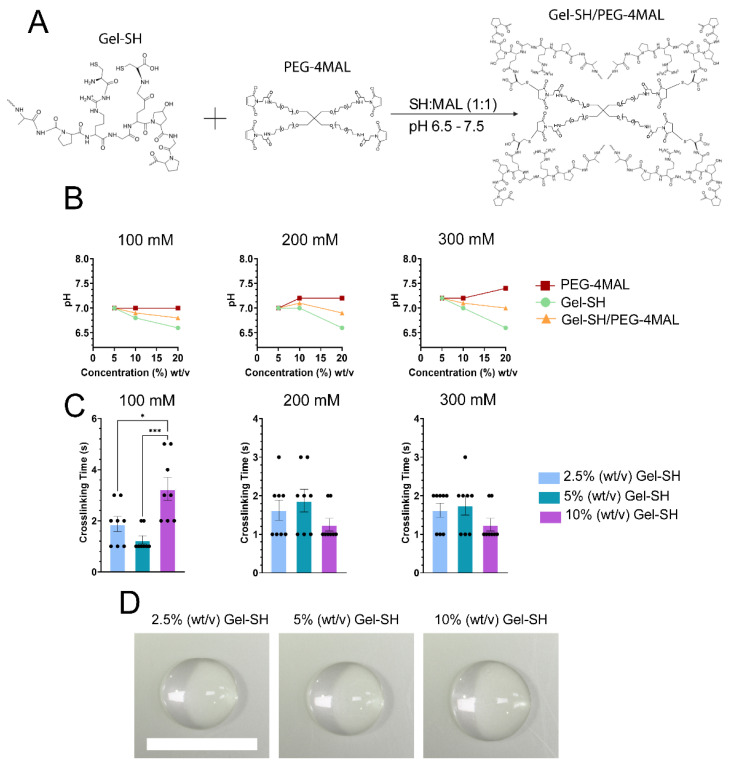
Gel-SH/PEG-4MAL click hydrogel reaction, crosslinking time and precursor pH using various concentrations of HEPES buffer. (**A**) Gel-SH/PEG-4MAL hydrogel crosslinking schematic. Gel-SH/PEG-4MAL hydrogels crosslink via Michael-type addition at a 1:1 SH:MAL molar ratio; (**B**) pH of hydrogel precursors and Gel-SH/PEG-4MAL hydrogels prepared using 100 mM, 200 mM and 300 mM HEPES buffer. (mean ± SEM; *n* = 3, * *p* ≤ 0.05, *** *p* ≤ 0.0005); (**C**) crosslinking times of Gel-SH/PEG-4MAL hydrogels. Crosslinking time was determined via pipette-mix method. Gel-SH concentration reported as final concentration within the hydrogel; (**D**) Gel-SH/PEG-4MAL hydrogels imaged via Nikon SMZ25 stereomicroscope. Scale = 1 cm.

After determining that Gel-SH solutions were highly functionalized and readily handled, their ability to undergo Michael-type addition with PEG-4MAL was investigated. Crosslinking between Gel-SH and PEG-4MAL is possible through Michael-type addition of Gel-SH thiol groups with the carbon-carbon double bond of the maleimide ring of PEG-4MAL, resulting in a stable iodosuccinimide bond (Figure 3A). The Michael-type reaction between the thiol group of Gel-SH and maleimide ring of PEG-4MAL is nucleophilic in nature; therefore, it was expected that the reaction kinetics may be influenced by solution pH [70]. Gel-SH and PEG-4MAL solutions were dissolved in 100 mM, 200 mM, and 300 mM HEPES buffer, to determine the effect of HEPES and hydrogel precursor concentration, on precursor and hydrogel pH, as well as hydrogel crosslinking time. HEPES buffer was chosen because of its optimal buffering within the pH range of Michael-type addition for thiol-maleimide conjugation (pH 6.5–7.5), as maleimides select for both thiols and amines when the reaction pH exceeds 7.5 [70,71].

As seen in Figure 3B, increasing the concentration of Gel-SH resulted in decreased solution pH for all concentrations of HEPES buffer tested, except for 100 mM HEPES, where increasing Gel-SH concentration did not alter the pH of the Gel-SH solution. Gel-SH pH decreased with increasing concentration most likely due to the acidic nature of the native gelatin [66,72,73]. The pH of the final Gel-SH/PEG-4MAL solutions fell within the range of optimal Michael-type addition [57,74], resulting in hydrogel crosslinking in less than four seconds for all HEPES conditions tested. All Gel-SH/PEG-4MAL hydrogels prepared using Gel-SH and PEG-4MAL solutions dissolved in 300 mM HEPES buffer crosslinked in less than two seconds of mixing (Figure 3C). The rapid crosslinking speed of hydrogels prepared using 300 mM HEPES buffer indicated that they would be advantageous for high-throughput, efficient preparation of hydrogels en masse, and opens up the potential for their use in applications such as drop-on-demand bioprinting, where fast crosslinking speed is desired [25,75]. For other applications, where slower crosslinking kinetics are desired, pH of the hydrogel precursors could be decreased [76] or other thiol-reactive crosslinkers, such as PEG-diacrylate [52,77], could be used in substitution of PEG-4MAL. Here, rapid crosslinking was desired for future applications in drop-on-demand bioprinting, therefore it was determined that 300 mM HEPES buffer was the most optimal buffer for rapid synthesis of Gel-SH/PEG-4MAL hydrogels.

Once Gel-SH/PEG-4MAL hydrogels were successfully prepared, their physical properties were characterized. Previous studies have demonstrated the tuneability of click based hydrogel system’s mechanical properties, such as the Young’s modulus (*E*) through the alteration of the hydrogel precursor concentrations [78,79]. Increasing the concentration of hydrogel precursors can increase the number of potential crosslinks between functional group within the final hydrogel matrix. Such crosslinks stabilize and strengthen the matrices [43]. As previously mentioned, matching hydrogel stiffness to that of in vivo tissues is of great importance for cell culture. Therefore, characterizing the physical and mechanical properties of hydrogels prepared at various concentrations will provide justification for their use with specific cell types, where the in vivo tissue stiffness is within the same range as that of the hydrogel being used. Therefore, it was hypothesized that Gel-SH/PEG-4MAL hydrogel storage modulus (*G′*) and Young’s modulus would increase with precursor concentration.

**Figure 4:**
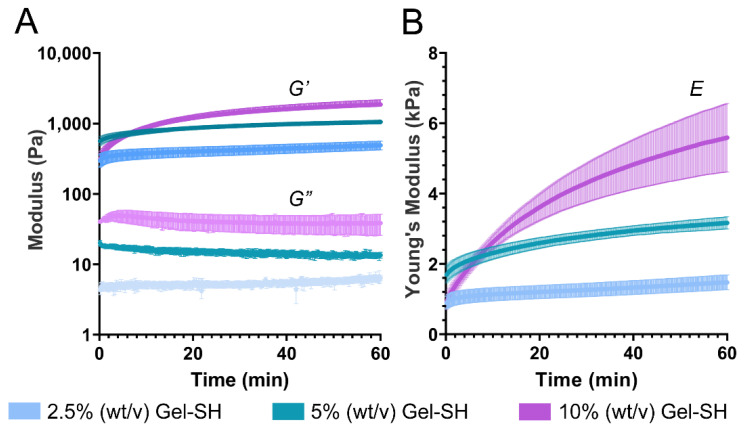
Rheological assessment of Gel-SH/PEG-4MAL hydrogel crosslinking kinetics and mechanical properties. (**A**) Storage modulus (*G′*) and loss modulus (*G″*) of Gel-SH/PEG-4MAL hydrogels over time (mean ± SEM; *n* = 3); (**B**) Young’s modulus (*E*) of Gel-SH/PEG-4MAL hydrogels over time. Young’s modulus was determined through Equation (1) using complex shear modulus and an assumed Poisson’s ratio of 0.5 (mean ± SEM; *n* = 3).

Through time sweeps of Gel-SH/PEG-4MAL hydrogels, matrix crosslinking was observed immediately post-crosslinking, as shown by storage modulus being greater than loss modulus (*G″*) for all conditions (Figure 4A). This was expected, based on the pipette-mix crosslinking test as seen in Figure 3C. Storage modulus continued to increase over time, with 2.5%, 5% and 10% (*w*/*v*) final Gel-SH Gel-SH/PEG-4MAL hydrogels possessing storage moduli of 492 Pa, 1055 Pa, and 1863 Pa, respectively, 1 h post-crosslinking. The storage and loss moduli of the 2.5% (*w*/*v*) final Gel-SH Gel-SH/PEG-4MAL hydrogels were similar to values reported by Utama et al. [25] for bis-thiol-PEG/PEG-4MAL hydrogels, where 5% (*w*/*v*) final PEG-4MAL bis-thiol-PEG/PEG-4MAL hydrogels possessed storage moduli of roughly 530 Pa. The maleimide-reactive hydrogel precursor used by Utama et al. was dissolved at ~1% (*w*/*v*); therefore, accounting for the final concentration of both Gel-SH and PEG-4MAL in the prepared hydrogels, the total concentration of 2.5% (*w*/*v*) final Gel-SH Gel- hydrogels is most similar to the hydrogels reported by Utama et al. These results indicated that although Michael-type addition between Gel-SH and PEG-4MAL forms 3D Gel-SH/PEG-4MAL matrices near-instantaneously, such matrices continue to crosslink over time.

**Figure 5:**
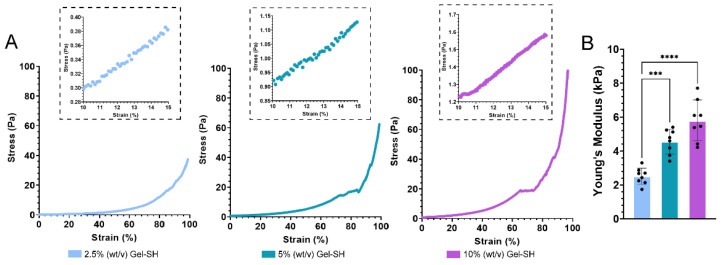
Compression testing of Gel-SH/PEG-4MAL hydrogels. (**A**) Stress–strain curves of Gel-SH/PEG-4MAL hydrogels, *n* = 1. (**B**) Young’s modulus of Gel-SH/PEG-4MAL hydrogels determined using hydrogel height, area, and slope of stress–strain curves at 10–15% strain (*n* = 8; mean ± SEM; *** = *p* ≤ 0.0001, **** = *p* ≤ 0.0001.

Additionally, rheology time sweeps showed that Gel-SH/PEG-4MAL Young’s moduli increased over time (Figure 4B), with Gel-SH/PEG-4MAL Young’s moduli 1 h post-crosslinking increasing with Gel-SH concentration. After one hour of crosslinking, 2.5% (*w*/*v*) final Gel-SH Gel-SH/PEG-4MAL hydrogels possessed Young’s moduli of 1.48 kPa, 5% (*w*/*v*) hydrogels possessed Young’s moduli of 3.17 kPa, and 10% (*w*/*v*) hydrogels possessed Young’s moduli of 5.59 kPa. This indicated that the mechanical stiffness of the hydrogels could be tuned by varying the precursor concentration. It should be noted that the time sweep method of determining Young’s moduli was conducted under the assumption that Poisson’s ratio of the hydrogels was 0.5, as is commonly used for gelatin-based hydrogel matrices [80]. Furthermore, hydrogel matrix mechanical properties may change due to swelling to equilibrium [77,81], which is difficult to capture using rheological analysis. Therefore, further mechanical analyses were conducted to determine Young’s moduli of Gel-SH/PEG-4MAL hydrogels using unconfined compression testing on hydrogels incubated in phosphate-buffered saline (PBS) at 37 °C overnight (Figure 5).

As seen in Figure 5, Gel-SH/PEG-4MAL hydrogel Young’s moduli increased with hydrogel concentration, as was expected based on the rheological assessment (Figure 4). Through compression testing, 2.5% (*w*/*v*) Gel-SH Gel-SH/PEG-4MAL hydrogels possessed an average Young’s moduli of 2.51 kPa, 5% (*w*/*v*) hydrogels possessed Young’s moduli of 4.56 kPa, and 10% (*w*/*v*) hydrogels possessed Young’s moduli of 5.80 kPa. For all conditions, hydrogel Young’s moduli appeared greater through compression testing compared to rheological assessment (Figure 4). As previously mentioned, matching in vivo stiffness is of great importance, especially when culturing cells that behave differently under various stiffness conditions. Therefore, the ability to tune the stiffness of Gel-SH/PEG-4MAL hydrogels should allow them to be used for the culture of multiple cell types with various stiffness requirements. Regarding the culture of breast cancer cells, through compression testing, Samani et al. found that breast fat and fibroglandular tissues exhibited Young’s moduli of 3.25 kPa, whereas breast cancer tumors possessed Young’s moduli ranging from 6.41 kPa to 42.51 kPa, depending on the type of tumor [82]. Using alternative methods of measurement, other studies have found that breast fibroglandular tissues typically possess Young’s moduli between 2.5 kPa and 3.5 kPa [83,84,85]. Therefore, Gel-SH/PEG-4MAL hydrogels would be suited for the culture of breast cancer cells, as the Young’s moduli of breast fat and fibroglandular tissues is similar to that of Gel-SH/PEG-4MAL hydrogels. Once it had been determined that Gel-SH/PEG-4MAL Young’s moduli could be tuned based on precursor concentration, swelling studies were conducted to determine whether changing precursor concentrations also affected the swelling characteristics of the hydrogels.

**Figure 6:**
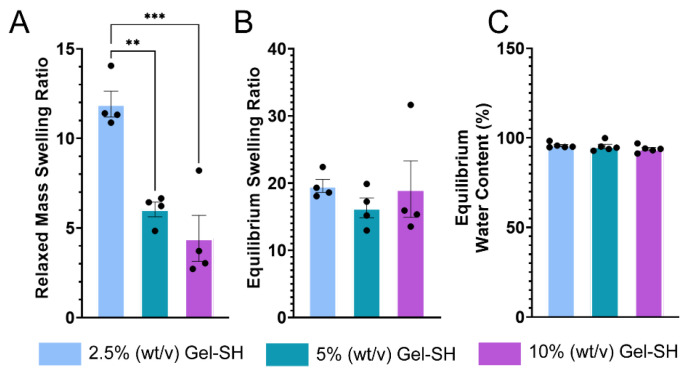
Swelling properties of Gel-SH/PEG-4MAL hydrogels. (**A**) Relaxed hydrogel swelling ratio (*Q_mr_*) describes the relationship between the weight of the dried hydrogel and weight of the hydrogel immediately after crosslinking. (**B**) Equilibrium swelling ratio (*Q_m_*) describes the relationship between the weight of the dried hydrogel and weight of the hydrogel after swelling. (**C**) Equilibrium water content (*EWC*) describes the capacity for the hydrogel to retain water when osmotic and ionic pressure of solutions external to the hydrogel matrix are at equilibrium with the pressure of the hydrogel matrix. (*n* ≤ 4; mean ± SEM; significance testing = one-way ANOVA; ** = *p* ≤ 0.001, *** = *p* ≤ 0.0001).

Hydrogels are porous, water-bound matrices, comprised of crosslinked biomaterials that possess various chain lengths and crosslinking densities. Through hydrophilic interactions of the constituent biomaterials’ functional groups, such as carboxyl and hydroxyl groups, hydrogels can swell when in the presence of water [47,86]. Hydrogel swelling is of great importance, as it directly relates to crosslinking density, which further informs the diffusion of molecules throughout the hydrogel matrix [87]. Molecular rate of diffusion is important in applications such as drug delivery, where differences in drug diffusion rate between models may be explained by variations in mesh size or crosslinking density [43,88]. Previous analyses of hydrogel pore-size have been conducted through the use of scanning electron microscopy (SEM); however, SEM was not used here due to limitations regarding sample preparation, as hydrogels must be lyophilized prior to SEM, therefore compromising the hydrogel’s structure, and may not accurately represent the true pore size of the hydrogel when swollen [89]. Here, Gel-SH/PEG-4MAL hydrogel relaxed swelling ratio (*Q_mr_*), equilibrium swelling ratio (*Q_m_*), and equilibrium water content (*EWC*), to determine the effect of Gel-SH/PEG-4MAL hydrogel concentration on swelling properties (Figure 6).

As seen in Figure 6, Gel-SH/PEG-4MAL *Q_mr_* decreased as hydrogel concentration decreased. This was to be expected, as *Q_mr_* is dependent on the weight of the hydrogels immediately after crosslinking. In contrast, *Q_m_* of Gel-SH/PEG-4MAL hydrogels was not dependent on hydrogel concentration. This was most likely due to the increase in hydrophilic side chains, such as carboxyl and hydroxyl groups [90,91], in hydrogels of increased concentration, that allow matrices to swell at a greater capacity once at equilibrium [87]. *EWC* describes the proportion of water in the hydrogel matrix when mechanical tension of the matrix, and the ionic and water osmotic pressure of surrounding media has reached equilibrium. Decreased *EWC* typically correlates with increased crosslinking density, as matrices with higher crosslinking densities can prevent swelling [87]. Here, no significant differences in *EWC* were observed between hydrogel concentrations, therefore indicating similar crosslinking densities between Gel-SH/PEG-4MAL hydrogels of different concentrations. The similar crosslinking densities between Gel-SH/PEG-4MAL hydrogel concentrations, indicated by *EWC*, indicate that increasing hydrogel concentration would not significantly impact diffusion of molecules throughout the matrices [92]; therefore, Gel-SH/PEG-4MAL hydrogels would not be limited in this manner for applications such as drug delivery [93].

Once the physical and mechanical properties of Gel-SH/PEG-4MAL hydrogels were characterized, we encapsulated MDA-MB-231 and MCF-7 breast cancer cell lines, to evaluate the potential for use of Gel-SH/PEG-4MAL as platforms for 3D cell culture. Previously, Gel-SH-based hydrogels have been used for 3D culture of murine adipose-derived stem cells [52], murine fibroblasts [57,94], neonatal human dermal fibroblasts [77] and hepatocellular carcinoma cells [95,96] however, Gel-SH-based hydrogel cell culture of cell types such as breast cancer have yet to be reported. Additionally, the previously reported Gel-SH formulations have been produced through functionalization of gelatin derived from mammalian sources, resulting in Gel-SH materials that possess high viscosity at room temperature, therefore increasing handling difficulty. The low-viscosity, ready-to-use nature of gelatin derived from cold-water fish skin [65,66,69], in addition to the rapid, non-laborious method of hydrogel preparation offered by Michael-type addition crosslinking, makes fish gelatin-based Gel-SH an attractive biomaterial for culture of cancer cells and primary cells. Breast cancer cells can organize differently depending on matrix stiffness, with some breast cancer cell lines, such as MCF-7, forming spheroids in lower stiffness matrices that match the stiffness of the in vivo breast microenvironment [33]. Spheroid formation can impact disease progression and drug resistance; therefore, it is ideal for 3D models to be able to recapitulate this characteristic in vitro, by matching in vivo stiffness. Additionally, the bone is a common metastatic site in breast cancer patients, and can cause skeletal-related events, such as spinal cord compression, and osteoporotic fractures to arise, impacting the quality of life and survival rate in breast cancer patients [97,98,99]. The skeleton is comprised of ~95% collagen, which gelatin is derived from. Therefore, Gel-SH-based hydrogels have potential for use as co-culture systems of both breast cancer and bone cells that can mimic this specific bone tumor microenvironment.

**Figure 7:**
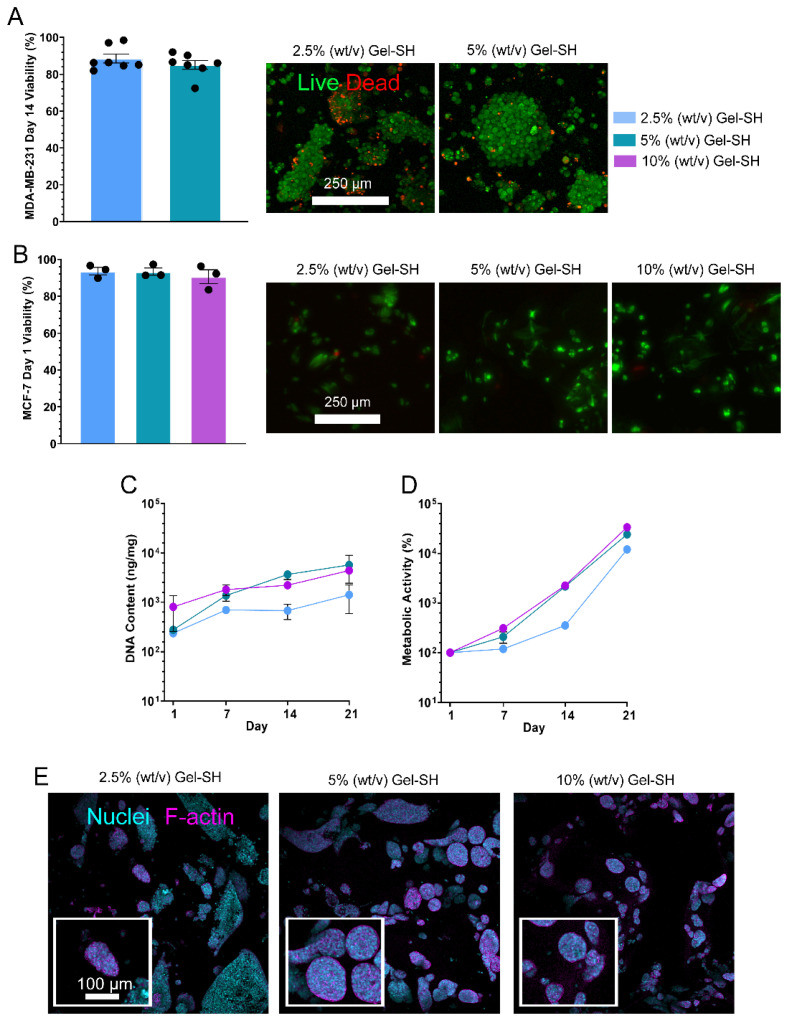
Growth of breast cancer cell lines in Gel-SH/PEG-4MAL hydrogels. (**A**) Day 14 viability of MDA-MB-231 cells encapsulated in Gel-SH/PEG-4MAL hydrogels, via FDA/PI live (green)/dead (red) fluorescent staining and confocal imaging (maximum projections of Z-stacks shown). Scale = 250 μm. (*n* = 7; mean ± SEM); (**B**) Day 1 viability of MCF-7 cells encapsulated in Gel-SH/PEG-4MAL hydrogels. Scale = 250 μm. (*n* = 3; mean ± SEM). (**C**) DNA content of MCF-7-encapsulated Gel-SH/PEG-4MAL hydrogels normalized to the wet weight of the hydrogels (*n* = 6; mean ± SEM); (**D**) Metabolic activity of MCF-7 cells encapsulated Gel-SH/PEG-4MAL hydrogels normalized to day 1 metabolic activity (*n* = 6; mean ± SEM); (**E**) Confocal imaging of MCF-7-encapsulated Gel-SH/PEG-4MAL hydrogels (blue = nuclei, magenta = f-actin). Scale = 100 μm.

To evaluate the potential of Gel-SH/PEG-4MAL hydrogels as platforms for 3D cell culture, an initial breast cancer cell viability study was conducted, where triple-negative breast cancer MDA-MB-231 cells were encapsulated in 2.5% (*w*/*v*) and 5% (*w*/*v*) final Gel-SH concentration Gel-SH/PEG-4MAL hydrogels, with viability assessment on day 14 post-encapsulation via live/dead staining. The viability of the MDA-MB-231 cells by day 14 post-encapsulation was high for both Gel-SH/PEG-4MAL hydrogel concentrations tested and cells formed grape-like clusters (Figure 7A), which has been observed in other 3D cell culture models [100,101]. After the pilot study, MCF-7 breast cancer cells were encapsulated in 2.5% (*w*/*v*), 5% (*w*/*v*) and 10% (*w*/*v*) Gel-SH/PEG-4MAL hydrogels, with an initial viability assessment 1-day post-encapsulation through live/dead staining (Figure 7B), and further DNA (Figure 7C) and metabolic activity assessments (Figure 7D) used in place of live/dead staining after day 1 due to MCF-7 spheroid formation (Figure 7E), that could prevent precise quantification of individual live and dead cells. Here, MCF-7 cells were chosen for extended culture due to their ability to form tumor-like spheroids, as seen in other gelatin-based 3D models such as GelMA hydrogels [102]. Breast cancer spheroids self-assemble through reorganization of the cytoskeleton, composed of microtubules, actin filaments and intermediate filaments, and intercellular interactions via cell tunneling nanotubes, amyloid fibrils, gap junctions, and cytoplasmic bridges [103,104]. MCF-7 spheroids resemble solid in vivo tumors physically and phenotypically, and as a result, present with similar drug resistance profiles [105,106], leveraging their potential as more accurate models for drug testing than cells grown in 2D [107,108,109].

As expected, based on the high viability of encapsulated MDA-MB-231 cells (Figure 7A), MCF-7s possessed high viability at day 1 of 3D cell culture through live/dead assessment (Figure 7B), and maintained viability over the 21-day period, as indicated by their increasing DNA content and metabolic activity over time (Figure 7C,D). Additionally, MCF-7 spheroid formation was observed by day 14 of culture (Figure 7E). This was expected based on the Young’s moduli of the Gel-SH/PEG-4MAL hydrogels (Figure 4 and Figure 5), where hydrogels possessed Young’s moduli similar to that of breast fat and fibroglandular tissue (roughly 2.5–3.5 kPa) [82,83,84,85]. From the conditions tested, it was observed that 5% (*w*/*v*) Gel-SH hydrogels provided the most suitable environment for spheroid formation, as spheroids appeared more frequently and greater in size in these hydrogels compared to 10% (*w*/*v*) and 2.5% (*w*/*v*) Gel-SH hydrogels. This is most likely because the Young’s moduli of the 5% (*w*/*v*) Gel-SH hydrogels (3.17 kPa (Figure 4) or 4.56 kPa (Figure 5), most similar to the previously reported range of Young’s moduli for patient breast fat and fibroglandular tissue. Overall, these findings suggest that the Gel-SH/PEG-4MAL hydrogels were suitable for 3D cell culture of breast cancer cell lines.

**Figure 8:**
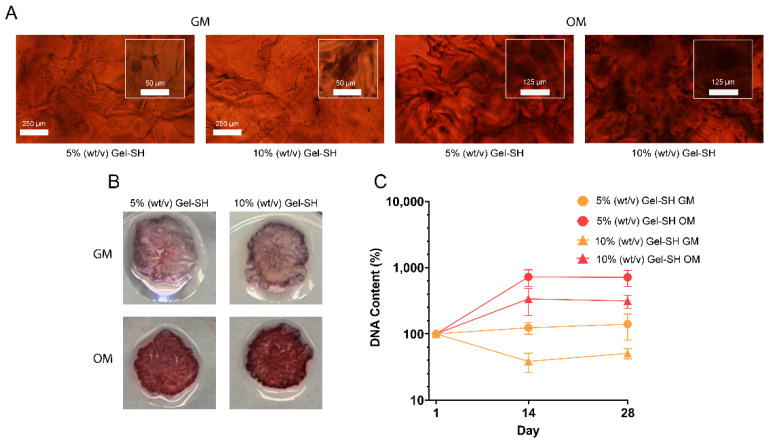
Osteogenic differentiation of patient-derived preosteoblasts in Gel-SH/PEG-4MAL hydrogels. (**A**) Day 28 alizarin red staining (ARS) of patient-derived preosteoblast-laden Gel-SH/PEG-4MAL hydrogels cultured using either α-MEM growth media (GM) or osteogenic media (OM). *n* = 6; (**B**) Day 28 macroscopic images of ARS-stained, patient-derived preosteoblast-laden Gel-SH/PEG-4MAL hydrogels; (**C**) DNA content of patient-derived preosteoblasts encapsulated in Gel-SH/PEG-4MAL hydrogels over time, normalized to hydrogel weight and day 1 DNA content (*n* ≤ 5; mean ± SEM).

After validating the Gel-SH/PEG-4MAL hydrogels as suitable platforms for culture of cell lines, the potential for Gel-SH/PEG-4MAL hydrogels to culture patient-derived cells was investigated. Patient-derived preosteoblasts were encapsulated into 5% (*w*/*v*) and 10% (*w*/*v*) final Gel-SH hydrogels, and cultured in either growth media (GM) or osteogenic media (OM) for 28 days, to determine whether Gel-SH/PEG-4MAL hydrogels could support the growth and proliferation of patient-derived cells, and support osteogenic differentiation of the preosteoblasts through the introduction of osteogenic conditions (Figure 8). Preosteoblast differentiation occurs in vivo during bone repair [110,111,112], where preosteoblasts are guided towards osteogenic cell lineages through specific environmental cues such as cytokines, growth factors, and nutrients, such as ascorbic acid, that hydroxylates proline residues, allowing collagen helical structure formation to occur [113]. Osteogenic differentiation results in the production of calcium, an inorganic mineral that comprises 65% of the human bone matrix [114], and can be targeted via alizarin red staining (ARS) [115]. Previous studies using 3D models, such as GelMA hydrogels, have used ARS to determine mineral deposition [116,117,118], and therefore, differentiation of preosteoblast cells into osteoblasts.

As seen in Figure 8A, patient-derived cells cultured in OM presented with a higher amount of mineralization compared to those cultured in GM, indicated by the increased presence of stained mineral deposits in OM cultures. Increased mineralization in OM cultures was also observed by macroscopic imaging of the hydrogels (Figure 8B). Additionally, DNA content of the encapsulated patient-derived cells increased from day 1 to day 14 when cultured in 5% (*w*/*v*) Gel-SH hydrogels in GM (124%) and OM (725%), and 10% (*w*/*v*) Gel-SH hydrogels in OM (338%) (Figure 8C). Interestingly, DNA content of patient-derived cells in 10% (*w*/*v*) Gel-SH hydrogels cultured in GM decreased from day 1 to day 14 (38%) but increased from day 14 to day 28 (51% of day 1). This, in conjunction with the observation that the DNA content of patient-derived cells increased more in 5% (*w*/*v*) and 10% (*w*/*v*) Gel-SH hydrogels cultured in OM than GM, showed that the patient-derived cells proliferated more in the osteogenic conditions provided by OM over the non-osteogenic conditions of GM. These results were similar to previous results described by Rajput et al., where MC3T3-E1 mouse calvarial preosteoblasts encapsulated in GelMA/silk fibroin hydrogels and cultured in OM exhibited darker ARS and higher DNA content than when cultured in GM [116]. Overall, these results suggest that the Gel-SH/PEG-4MAL hydrogels are suitable platforms for culture of patient-derived cells and provide environments that support osteogenic differentiation. These findings, in conjunction with the high viability of encapsulated breast cancer cell lines (Figure 7), display the potential of Gel-SH/PEG-4MAL hydrogels for future use as co-culture models for the study of breast-to-bone cancer metastasis.

## 3. Conclusions

A novel, low-viscosity biomaterial, Gel-SH, alongside Gel-SH/PEG-4MAL hydrogels, have been successfully synthesized and characterized. The evaluation of the chemical properties of Gel-SH confirmed the successful functionalization of the raw gelatin biomaterial with thiol functional groups, that could readily undergo Michael-type click crosslinking with PEG-4MAL to form Gel-SH/PEG-4MAL hydrogels. Rheological characterization demonstrated Newtonian-like flow characteristics in all Gel-SH concentrations independent of temperature. Through click crosslinking, Gel-SH/PEG-4MAL hydrogels were successfully prepared in less than five seconds for each condition tested when precursors were dissolved in 300 mM HEPES buffer. Rheological and compression testing of the hydrogels showed that their mechanical stiffness could be tuned through the alteration of precursor concentration, and that their stiffness was within the range of native and cancerous breast tissue. Additionally, through swelling studies, it was determined that the crosslinking density of the hydrogels did not change depending on hydrogel concentration, therefore indicating that molecular diffusion and drug treatment would not be impeded by alteration of hydrogel concentration. Assessment of the breast cancer and patient-derived preosteoblast cells encapsulated in Gel-SH/PEG-4MAL hydrogels showed that the hydrogels were valid platforms for the culture of breast cancer cell lines and patient-derived cells and have potential for future use as co-culture models for breast-to-bone cancer metastasis models.

## 4. Materials and Methods

*Synthesis of Gel-SH.* 10 g gelatin from cold-water fish skin (Sigma^®^, Castle Hill, Australia, Lot #SLCG7135) was added to 500 mL 0.1 mM HCl and stirred at RT until dissolved. Then, 7.5 g 1-ethyl-3(3-dimethylamino)propyl carbodiimide (EDC) (Sigma^®^, Castle Hill, Australia) and 3.75 g *N*-hydroxysuccinimide (NHS) (Sigma^®^, Castle Hill, Australia) were added to the solution. The EDC/NHS reaction was allowed to continue for 30 min, then, 20 g L-cysteine (Sigma^®^, Castle Hill, Australia) was added to the solution. The conjugation reaction proceeded for 24 h at room temperature protected from light. The pH of the solution was maintained at 5.0 throughout the reaction. Then, the solution was dialyzed against 0.1 mM HCl using 1 kDa molecular weight cut-off snakeskin dialysis tubing (Thermo Scientific™, Waltham, MA, USA) for 5 days. Once dialysis was complete, samples were frozen overnight at −80 °C and lyophilized for 5 days.

*TNBS Assay.* The amine content of Gel-SH was quantified via 2,4,6-trinitrobenzenesulfonate (TNBS) assay, as described by Pahoff et al. [117]. Briefly, 0.1 M NaHCO_3_ buffer was prepared, and pH of the solution was adjusted to 8.5 using HCl and NaOH. A 0.01% (*w*/*v*) TNBS solution was prepared by 1:500 dilution of TNBS stock. Gel-SH and gelatin from cold-water fish skin were then dissolved in 0.1 M NaHCO_3_ buffer at 10 mg/mL. A volume of 250 μL of each solution was diluted to 500 μg/mL using 0.1 M NaHCO_3_ buffer. A 1:2 dilution series of Gel-SH and gelatin from cold-water fish skin was prepared with concentrations ranging from 0–500 μg/mL. An L-cysteine standard dilution series of 0.5–0.156 mM was prepared. A volume of 200 μL of each sample and standard dilution was added in triplicate to a clear 96-well plate (Corning^®^ Costar^®^, New York, NY, USA), and 100 μL 0.01% (*w*/*v*) TNBS solution was added. Samples were then mixed on a plate shaker for 5 min protected from light. Then, samples were transferred to a 37 °C laboratory oven and incubated for 2 h protected from light. Well-plate absorbance was read at 335 nm using a CLARIOstar^®^ (BMG Labtech, Mornington, Australia) spectrophotometer. The amine content of the samples was determined through comparison of sample absorbance to the absorbance of the L-cysteine standard curve.

*DTNB Assay.* The thiol content of Gel-SH was quantified via 5,5′-Dithio-bis-(2-nitrobenzenoic acid, DTNB) assay, as previously described [59]. A 1:2 L-cysteine standard dilution series of 0–2 mM was prepared. Gel-SH and gelatin from cold-water fish skin were dissolved at 5 mg/mL in PBE. A 1:2 dilution series of samples was prepared with concentrations ranging from 500 μg/mL to 125 μg/mL. A volume of 25 μL of each sample and standard was then added in triplicate to a clear 96-well plate (Corning^®^ Costar^®^, New York, NY, USA). A volume of 125 μL of DTNB solution was then added to each sample. The well-plate was then shaken on a plate shaker and incubated at RT protected from light for 15 min. Post-incubation, the absorbance of the well-plate was measured at 412nm using a CLARIOstar^®^ (BMG Labtech, Mornington, Australia) well-plate reader. The thiol content of Gel-SH was determined through comparison of sample absorbance to the absorbance of the L-cysteine standard curve.

*^1^H-NMR.* Proton nuclear magnetic resonance (^1^H-NMR) was conducted to characterize the molecular profile of Gel-SH. Gel-SH and gelatin from cold-water fish skin were dissolved in 90% H_2_O/10% D_2_O solution, to a final concentration of 1% (*w*/*v*). A volume of 1 mL of each sample was added to respective NMR tubes and sample spectra were collected using a Bruker Avance 600 MHz (Bruker, Billerica, MA, USA) NMR instrument with water suppression. The sample spectra were analyzed using Bruker TopSpin 3.6.4.

*Rheology.* The rheological properties of Gel-SH and Gel-SH/PEG-4MAL hydrogels were determined using an Anton-Paar modular compact rheometer (MCR) 302 (Anton-Paar, Graz, Austria). Shear-rate sweeps were conducted at 25 °C using a 25 mm cone plate (CP25), with shear-rate range of 0.1–1000 per second, at a constant frequency of 1 Hz. Temperature sweeps were conducted using a 25 mm parallel plate (PP25) at a constant frequency of 1 Hz, and constant strain of 1%, with temperature ramping linearly from 37 °C to 0 °C, at a rate of 2 °C/min. Time sweeps were conducted using a PP25 at a constant frequency of 1 Hz and constant strain of 1%, for 1 h. Young’s modulus (*E*) of Gel-SH/PEG-4MAL hydrogels determined through Equation (1) as reported previously [25,118]:

(1) E=2G×(1+v)

where *G* is the complex shear modulus; and *v* is the assumed Poisson’s ratio (0.5) [80].

*Gel-SH/PEG-4MAL Hydrogel Preparation, Crosslinking Time, and pH.* Gel-SH and PEG-4MAL (MW 20 kDa, JenKem^®^, Plano, TX, USA) were dissolved in 300 mM, 200 mM and 100 mM HEPES buffer (Gibco™, Thermo Scientific™, Waltham, MA, USA, Lot #2185833) at 20% (*w*/*v*), 10% (*w*/*v*) and 5% (*w*/*v*), respectively. Equimolar Gel-SH/PEG-4MAL hydrogels with a 10% (*w*/*v*) final Gel-SH concentration were prepared by addition of 10 μL 20% (*w*/*v*) Gel-SH precursor solution to 10 μL 20% (*w*/*v*) PEG-4MAL precursor solution in 96-well plates (Corning^®^, New York, NY, USA). Crosslinking time was determined via pipette mixing, where time of crosslinking was defined as the time at which hydrogel solution could no longer be pipetted. 5% (*w*/*v*) and 2.5% (*w*/*v*) final Gel-SH concentration Gel-SH/PEG-4MAL hydrogels were prepared through the method above using 10% (*w*/*v*) and 5% (*w*/*v*) hydrogel precursors, respectively. The pH of each hydrogel precursor and hydrogel was determined through addition of 1 μL hydrogel precursor or hydrogel to 5.5–8.0 pH test strips (Hydrion^®^, New York, NY, USA).

*Compression Testing.* 10% (*w*/*v*), 5% (*w*/*v*) and 2.5% (*w*/*v*) final Gel-SH Gel-SH/PEG-4MAL hydrogels were prepared using 300 mM HEPES buffer and swollen overnight in phosphate-buffered saline (PBS) at 37 °C in a cell culture incubator. Prior to compression testing, hydrogels were imaged using a Nikon^®^ SMZ25 stereomicroscope (Nikon^®^, New York, NY, USA), and surface area was determined using ImageJ software (version 1.52a, National Institute of Health (NIH), USA). Hydrogels were then submerged in a PBS-filled water bath at 37 °C and compressed in an unconfined configuration using an Instron 5567 Microtester (Instron^®^, Norwood, MA, USA) with 5 N load cell (Instron^®^, Norwood, MA, USA) and non-porous aluminium indenter, at a strain rate of 0.01 mm/s. Young’s moduli (*E*) of the hydrogels were determined as the slope of stress–strain curves at 10–15% strain.

*Equilibrium Swelling and Mass Swelling Ratios.* 10% (*w*/*v*), 5% (*w*/*v*) and 2.5% (*w*/*v*) final Gel-SH hydrogels *n* ≤ 4 were prepared using 300 mM HEPES buffer and weighed immediately post-crosslinking. Hydrogels were then swollen overnight in PBS at 37 °C overnight. After swelling, hydrogels were re-weighed, then lyophilised. The recorded weights of the hydrogels were used to calculate the equilibrium water content (*EWC*) using Equation (2), as reported previously [87]:

(2) $$EWC\;\left(\%\right)=\frac{\left(m_{wet}-m_{lyophilized}\right)}{m_{wet}}\times 100\;$$

where *m_wet_* is the mass of hydrogel post-swelling, and *m_lyophilised_* is the mass of hydrogel after lyophilization.

The equilibrium mass swelling ratio (*Q_m_*) was calculated using Equation (3):

(3) $$Q_{m=}\frac{\left(m_{wet}-m_{lyophilized}\right)}{m_{wet}}\;$$

The relaxed mass swelling ratio (*Q_mr_*) was determined using Equation (4):

(4) $$Q_{mr=}\frac{\left(m_{crosslinked}-m_{lyophilized}\right)}{m_{crosslinked}}\;$$

where *m_crosslinked_* is the mass of hydrogel immediately post-crosslinking.

*Cell Culture.* MDA-MB-231 breast cancer cells (ATCC, passage #9) were seeded in T175 flasks (Nunc^®^, Thermo Scientifc™, Waltham, MA, USA) and cultured in Dulbecco’s Modified Eagle Medium (DMEM, Thermo Scientific™, Waltham, MA, USA) supplemented with 10% (*v*/*v*) foetal bovine serum (FBS), 1% (*v*/*v*) penicillin streptomycin (P/S) and 1% (*v*/*v*) glutamine in cell culture incubators at 37 °C with complete media changes every three–four days until 70–80% confluency was achieved. MCF-7 breast cancer cells (ATCC, passage #3) were cultured in RPMI 1640 medium (Gibco™, Thermo Scientific™, Waltham, MA, USA) supplemented with 10% (*v*/*v*) FBS, 1% (*v*/*v*) P/S, 1% (*v*/*v*) non-essential amino acids, 1% (*v*/*v*) sodium pyruvate, and 0.1% (*v*/*v*) insulin-transferring-selenium, and incubated in cell culture incubators at 37 °C, 5 % CO2 with complete media changes every three–four days until 70–80% confluency was achieved. Patient-derived preosteoblasts (passage #3) were obtained from female donors undergoing hip and knee replacement surgery, under QUT ethics approval # 1400001024, as described previously [36]. Cells were seeded in T175 flasks and cultured in growth media (GM) (αMEM, Thermo Scientific™, Waltham, MA, USA) supplemented with 10% (*v*/*v*) FBS and 1% (*v*/*v*) P/S in cell culture incubators at 37 °C, 5% CO2 with media changes every three–four days until reaching 90% confluency.

*Encapsulation of Cells in Gel-SH/PEG-4MAL Hydrogels*. Stock solutions of 20% (*w*/*v*), 10% (*w*/*v*), and 5% (*w*/*v*) Gel-SH and PEG-4MAL were prepared in 300 mM HEPES buffer. Cells were lifted using 0.25% Trypsin/ethylenediaminetetraacetic acid (EDTA, Thermo Scientific™, Waltham, MA, USA) and counted. MDA-MB-231 and MCF-7 cell lines were resuspended in Gel-SH at a concentration of 2 × 10^6^ cells/mL, patient-derived preosteoblasts were resuspended in Gel-SH at a concentration of 4 × 10^6^ cells/mL. A volume of 10 μL PEG-4MAL solution was added to 48-well plates (Corning^®^, New York, NY, USA), then, 10 μL of Gel-SH cell suspension was pipette-mixed with PEG-4MAL solution until crosslinking occurred. Post-crosslinking, cell-laden hydrogels were incubated in 1 mL of either RPMI media (MDA-MB-231 and MCF-7-laden hydrogels) or α-minimal essential growth media (GM, patient-derived preosteoblast-laden hydrogels) at 37 °C in cell incubators. Then, four days after encapsulation of patient-derived preosteoblast cells, GM was replaced with osteogenic media (OM), consisting of GM supplemented with 1 M β-glycerophosphate (Sigma-Aldrich™, Castle Hill, Australia), 0.1 M ascorbate-2-phosphate (Sigma-Aldrich™, Castle Hill, Australia), and 0.1 M dexamethasone (Sigma-Aldrich™, Castle Hill, Australia).

*Day 1 Viability.* The viability of cells encapsulated in Gel-SH/PEG-4MAL hydrogels one day post-encapsulation was determined using fluorescein diacetate (FDA) (Thermo Scientific™, Waltham, MA, USA)/propidium iodide (PI) (Thermo Scientfic™, Waltham, MA, USA) assay. Cell media were aspirated, and samples were washed with PBS at room temperature for 5 min, then incubated with staining solution (10 μg/mL FDA and 5 μg/mL PI in PBS) for 2 min. The staining solution was aspirated, and samples were washed for 2 min in PBS. The samples were then transferred to a glass slide and imaged using either a Leica SP5 confocal microscope for MDA-MB-231 cells, or Nikon^®^ SMZ25 epifluorescent microscope (Nikon^®^, New York, NY, USA) for MCF-cells. Z-stacks of hydrogels were captured with 10 μm slice intervals, and maximum intensity projections of hydrogel Z-stacks were obtained using ImageJ. Cell viability was determined through quantification of particles in live and dead channels of maximum intensity projections.

*Metabolic Activity.* The metabolic activity of cells encapsulated in Gel-SH/PEG-4MAL hydrogels was determined via PrestoBlue™ (Thermo Scientific™, Waltham, MA, USA) assay following the manufacturer’s instructions. Briefly, PrestoBlue™ staining solution was prepared by addition of 90% cell media (RPMI for MDA-MB-231 and MCF-7-laden hydrogels, GM for patient-derived preosteoblast-laden hydrogels) to 10% (*v*/*v*) PrestoBlue™ stock reagent. Hydrogels were washed with PBS for 5 min at room temperature, followed by 500 μL PrestoBlue™ staining solution to each well. Hydrogels were then incubated at cell culture incubator for 45 min. Following incubation, 100 μL PrestoBlue™ solution was aspirated from wells and added in triplicate to 96-well plates (Corning^®^, New York, NY, USA). The fluorescence of the well-plates was read at an excitation wavelength of 540 nm and emission wavelength of 590 nm using a CLARIOstar^®^ Plus spectrophotometer (BMG Labtech, Mornington, Australia). The metabolic activity of 3D cell cultures was normalized against day 1 metabolic activity.

*DNA Content*. The DNA content of cells encapsulated in Gel-SH/PEG-4MAL hydrogels was determined via Quant-iT™ PicoGreen^®^ (Life Technologies, Carlsbad, CA, USA) assay. Cell-laden hydrogels were transferred to 2 mL centrifuge tubes and frozen at −80 °C. Samples were then incubated in 500 μL proteinase K solution overnight at 65 °C in a heating block. DNA standards were prepared with concentrations ranging from 2000 ng/mL to 31.25 ng/mL. Samples were diluted in PBE and transferred in triplicate to a black 96-well plate. An amount of 100 μL of PicoGreen^®^ dye was (Life Technologies, Carlsbad, CA, USA) added to each well, and the well-plate was incubated for 5 min at room temperature protected from light. Post-incubation, the fluorescent signal of the samples and standards was measured at an excitation wavelength of 480 nm and emission wavelength of 520 nm, using a CLARIOstar^®^ Plus spectrophotometer (BMG Labtech, Mornington, Australia). The DNA content of sample digests was determined by comparison of sample fluorescence to DNA standard curve and normalized against hydrogel weight.

*Immunofluorescence staining.* Staining of the nuclei and f-actin filaments of MCF-7 cells encapsulated in Gel-SH/PEG-4MAL hydrogels was conducted using diamidino-2-phenylindole (DAPI) (Thermo Scientific™, Waltham, MA, USA)/Alexa-Fluor™ (Thermo Scientific™, Waltham, MA, USA) 488-conjugated phalloidin stains. On days 1, 7, 14 and 21, media were removed from MCF-7 hydrogel wells. Hydrogels were then washed with 1 mL PBS for 10 min at RT. Then, PBS was aspirated, and samples were fixed in 1 mL 4% (*w*/*v*) paraformaldehyde (PFA) for 1 h. Post-fixing, PFA was aspirated, and hydrogels were washed with 1 mL PBS. PBS for washing was aspirated, and an additional 1 mL aliquot of PBS was added to the hydrogels. Hydrogels were then stored at 4 °C. PBS was aspirated from wells and hydrogels were blocked using 300 μL blocking buffer (5% (*v*/*v*) goat serum (Gibco™, Thermo Scientific™, Waltham, MA, USA), 0.1% (*v*/*v*) Triton X-100 (Sigma™, Castle Hill, Australia) per hydrogel overnight on plate shaker at 4 °C. The blocking buffer was then aspirated, and hydrogels were washed twice with PBS at RT for 5 min each wash. PBS was aspirated and 150 μL of 1:1000 DAPI, 1:200 phalloidin in PBS was added to each hydrogel. The hydrogels were incubated at 4 °C overnight on plate shaker. Post-incubation, the staining solution was aspirated, and hydrogels were washed with 300 μL washing buffer (20% (*v*/*v*) blocking buffer, 1% (*v*/*v*) goat serum) per hydrogel three times over the course of 8 h at 4 °C on plate shaker. Post-washing, washing buffer was aspirated and hydrogels were washed with PBS three times and stored at 4 °C until imaging. The hydrogels were imaged using Leica SP5 confocal microscope at 4× objective. The DAPI channel was captured at an excitation wavelength of 405 nm, and phalloidin was captured at an excitation wavelength of 488 nm.

*Alizarin Red Staining*. On days 1, 14, and 28 post-OM addition to preosteoblast-laden Gel-SH/PEG-4MAL hydrogels, cell media were aspirated from 3D cultures, and samples were washed twice with PBS. Samples were then fixed in 300 μL ice-cold methanol for 10 min at room temperature. Once fixed, methanol was aspirated, and hydrogels were washed twice with ultrapure water. The samples were then incubated in 100 μL 1% (*w*/*v*) alizarin red staining solution (ARS) (Sigma™, Castle Hill, Australia). After incubation, samples were washed with ultrapure water 6 times, at which point the sample solutions had become clear. The samples were imaged using an Olympus IX73 brightfield microscope (Olympus, Shinjuku, Japan), and plates were stored at 4 °C.

## 5. Patents

The work reported in this manuscript has resulted in patent number No. 2022903674.

## Data Availability

All data needed to evaluate the conclusions in the paper are present in the paper. Additional data related to this paper may be requested from the authors, upon reasonable request.
